# Antioxidant, Tyrosinase, α-Glucosidase, and Elastase Enzyme Inhibition Activities of Optimized Unripe Ajwa Date Pulp (*Phoenix dactylifera*) Extracts by Response Surface Methodology

**DOI:** 10.3390/ijms24043396

**Published:** 2023-02-08

**Authors:** Fanar Alshammari, Md Badrul Alam, Bo-Rim Song, Sang-Han Lee

**Affiliations:** 1 Department of Food Science and Biotechnology, Graduate School, Kyungpook National University, Daegu 41566, Republic of Korea; 2 Food and Bio-Industry Research Institute, Inner Beauty/Antiaging Center, Kyungpook National University, Daegu 41566, Republic of Korea

**Keywords:** Ajwa date, enzymatic activity, polyphenolics, response surface methodology

## Abstract

The Ajwa date (*Phoenix dactylifera* L., Arecaceae family) is a popular edible fruit consumed all over the world. The profiling of the polyphenolic compounds of optimized unripe Ajwa date pulp (URADP) extracts is scarce. The aim of this study was to extract polyphenols from URADP as effectively as possible by using response surface methodology (RSM). A central composite design (CCD) was used to optimize the extraction conditions with respect to ethanol concentration, extraction time, and temperature and to achieve the maximum amount of polyphenolic compounds. High-resolution mass spectrometry was used to identify the URADP’s polyphenolic compounds. The DPPH-, ABTS-radical scavenging, α-glucosidase, elastase and tyrosinase enzyme inhibition of optimized extracts of URADP was also evaluated. According to RSM, the highest amounts of TPC (24.25 ± 1.02 mgGAE/g) and TFC (23.98 ± 0.65 mgCAE/g) were obtained at 52% ethanol, 81 min time, and 63 °C. Seventy (70) secondary metabolites, including phenolic, flavonoids, fatty acids, and sugar, were discovered using high-resolution mass spectrometry. In addition, twelve (12) new phytoconstituents were identified for the first time in this plant. Optimized URADP extract showed inhibition of DPPH-radical (IC_50_ = 87.56 mg/mL), ABTS-radical (IC_50_ = 172.36 mg/mL), α-glucosidase (IC_50_ = 221.59 mg/mL), elastase (IC_50_ = 372.25 mg/mL) and tyrosinase (IC_50_ = 59.53 mg/mL) enzymes. The results revealed a significant amount of phytoconstituents, making it an excellent contender for the pharmaceutical and food industries.

## 1. Introduction

Antioxidative phenolics found in the tissues of many plant species are thought to be responsible for their medicinal actions. They play a variety of purposes in plants, from structural to defensive [1]. However, studies have demonstrated phenolics’ preventive significance in diabetes, chronic cardiovascular illnesses, cancer, and aging cases [2,3]. Their positive effects on human health have thus far undergone substantial study. The study of polyphenolic compounds is gaining popularity, and the first and most crucial stage in extracting and purifying polyphenolic compounds from plant sources is extraction [4], given that the extraction of polyphenol is influenced by several factors, including the chemical makeup of the sample, the solvent employed, agitation, extraction time, solute/solvent ratio, and temperature [5,6]. Furthermore, phenolic molecules should not be oxidized because they participate in the enzymatic browning reaction and lose their phenol activity and antioxidant capacity [7]. Additionally, phenolic compounds’ structural and physicochemical diversity precludes a uniform extraction methodology and necessitates a unique strategy for each phenolic source [7]. Therefore, it is essential to research extraction conditions to enhance polyphenolic compound yield.

Tyrosinase is the type-3 metalloenzyme most closely related to the formation of melanin [8]. Living organisms naturally produce melanin to protect the skin from UV rays and reactive oxygen species (ROS). Wrinkles and skin hyperpigmentation brought on by too much melanin are urgent problems in the cosmetics industry [9]. Tyrosinase activity modulation has been the main focus of control measures for melanin formation. Because of their structural similarities to the enzyme’s substrate, L-tyrosine, polyphenolic compounds are the source of most tyrosinase inhibitors [9,10]. Furthermore, α-glucosidase is one of the essential enzymes for diabetes mellitus (DM). α-glucosidase hydrolyzes the 1,4-glucosidic bonds of oligosaccharides to create monosaccharides, which are absorbed into the blood from the intestine [11]. As a result, inhibitors of α-glucosidase can significantly lower postprandial hyperglycemia following a mixed-carbohydrate diet and may be used to manage DM. Furthermore, human neutrophil elastase (HNE) is a serine protease with a single polypeptide chain that is stored and secreted by polymorphonuclear neutrophils. It is a member of the elastase-like serine proteases subfamily [12]. Excess extracellular HNE, which can break down structural proteins of the extracellular matrix such as elastin, proteoglycan, collagen, and fibronectin, is brought on by imbalances between NE and its endogenous inhibitors [13]. NE can destroy elements of the coagulation and fibrinolytic pathways, as well as activate matrix metalloproteinases and deactivate their inhibitors. Following this, an excess of HNE may cause a number of pathological illnesses and tissue damage, including rheumatoid arthritis, psoriasis, cystic fibrosis, chronic obstructive lung disease, acute respiratory distress syndrome, pulmonary fibrosis, and pulmonary fibrosis [14,15,16,17]. The ability of the serine protease inhibitors to control the proteolytic activities of the serine proteases makes them vital for restoring the balance between the protease and anti-protease systems, limiting excessive elastin proteolysis, and lowering neutrophil accumulation at inflammatory areas [13,15]. Natural compounds such as polyphenolic compounds (ugonins Q: IC_50_ = 0.49 μM, quercetin-3-O-glucoside; IC_50_ = 0.35 μM, 6,8-diprenylorobol; IC_50_ = 1.3 μM, and amentoflavone; IC_50_ = 1.27 μM) are primarily found in herbal plants and have been shown to affect elastase release [12,14].

The extraction of phenolic chemicals must be optimized to produce a reliable result. It is generally possible to optimize a process using either empirical or statistical methods. The empirical one-factor-at-a-time technique includes altering one component at a time while keeping the other variables constant [18]. This approach’s fundamental flaw is that it ignores how the variables interact, making it impossible to account for all of a parameter’s impacts on the response. Another burden is that it takes many trials to complete the investigation, which extends the time, expense, reagent, and material consumption [18]. To overcome this challenge, multivariate statistical methods were used to optimize the analytical processes. The response surface methodology (RSM) is one of the most well-known multivariate approaches used in analytical optimization. Intending to optimize the desired response, RSM is a set of statistical and mathematical approaches for creating, developing, and modifying procedures where several variables have an impact. In addition to improving the design of existing products, it can be used to develop, formulate, and build new ones. It explains how the independent variables might affect the processes individually or collectively. In addition to evaluating the effects of independent components, this experimental approach offers a mathematical model that illustrates the chemical or biological processes [18,19].

Ajwa dates (*Phoenix dactylifera* L., Arecaceae family) are only cultivated in Madinah, Saudi Arabia, and are a popular edible fruit consumed worldwide. It is one of the market’s most expensive and valued cultivars owing to ethnomedical beliefs regarding its health-promoting qualities [20]. It is regarded to have cardioprotective [21], hepatoprotective [22], nephroprotective [23] and constipation-relieving [24] properties and antioxidant, anti-inflammatory, anticancer [25], antifungal, antibacterial, and antiviral activities [26]. In addition, it contains abundant bioactive components such as polyphenols, including phenolic acids, flavonoids, and lignans [20].

To the best of our knowledge, this is the first report that uses RSM to improve the extraction conditions so that more polyphenolic components may be extracted from the pulp of unripe Ajwa dates (URADP). The goal was to obtain the highest polyphenolic content possible from URADP by investigating and optimizing extraction parameters such as extraction temperature and duration, as well as ethanol concentration, using the RSM central composite design (CCD) tool. The RSM-CCD approach’s projected values accurately reflect the actual findings, and this statistical technique can be used to maximize the extraction of URADP polyphenolic compounds.

## 2. Results and Discussion

Response surface methodology (RSM) is a collection of mathematical and statistical methods built on fitting polynomial equations to experimental data. It accurately describes the behavior of data collection designed to produce statistical predictions. It is better than traditional single-parameter optimization since it takes less time, space, and raw materials [18,19].

Scientific information dealing with optimization of the extraction of polyphenols from unripe Ajwa date pulp (URADP) extracts is very inadequate. Mounting evidence has revealed the optimization of ultrasonic assistance extraction, microwave-assisted extraction, and supercritical fluid extraction procedures that were performed to extract polyphenols from different varieties of dates except from Ajwa dates [4,27,28,29]. To the best of our knowledge, this is the first report dealing with the optimization of heat extraction on individual biologically active polyphenols as dependent variables.

### 2.1. Fitting of the RSM Models

Table 1 lists the experimental conditions and findings for each extraction scenario. All response variables were transformed into second-order quadratic polynomials to account for extraction factor effects. The statistical significance of the fitted second-order quadratic model equations was assessed using ANOVA. The fitness of the model was evaluated using the regression coefficient (β), adjusted correlation factor (R^2^), coefficient of variation (CV), and adequate precision (Table 2). The non-significant terms (*p >* 0.05) were removed to enhance the models’ fit and predictions. *p* values were used to assess each coefficient’s significance. The model terms were statistically significant, extremely significant, and impressively significant when the *p* values were less than 0.05, 0.01, and 0.001, respectively.

**Figure 1:**
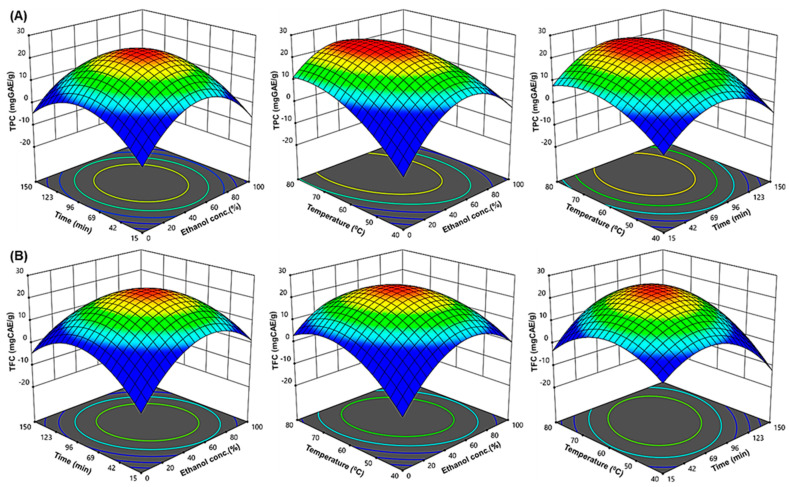
The three-dimensional (3D) response surface plots of URADP extraction for (**A**) TPC and (**B**) TFC for ethanol concentration, time, and temperature as a function of key interaction factors for RSM.

From Table 2, smaller probability values (*p* < 0.0001) indicate that the model terms are significant. In general, proceeding with exploration and optimization of a fitted response surface may produce poor or misleading results unless the model exhibits an adequate fit [7]. The developed regression models have a high degree of statistical significance, as indicated by their R^2^ values (0.9706 and 0.9968). The appropriate precision value is an indicator of the signal-to-noise ratio. It is preferable to have a ratio of >4 [25]. Here, the ratios were 15.9930 and 49.6969, suggesting a sufficient signal, indicating that the model is suitable for this procedure. The coefficient of variation (CV) is a measure of a model’s reproducibility and describes the extent to which the data were dispersed. The CV for total phenolic content (TPC) and total flavonoid content (TFC) of URADP was within the acceptable range (Table 2). Since CV is a measure expressing standard deviation as a percentage of the mean, the small values of CV give better reproducibility. In general, a high CV indicates that variation in the mean value is high and does not satisfactorily develop an adequate response mode [7]. The modified R^2^ (R^2^ ≥ 0.80) was well within acceptable limits in this study, showing that the experimental data fit second-order polynomial equations satisfactorily. To demonstrate the interactions between the independent variables, 3D surfaces and contour plots were constructed using multiple linear regression equations. The main and cross-product effects of the independent variables on the response variables are more easily understood from these 3D charts (Figure 1A,B).

### 2.2. Effect of Extraction Parameters on TPC and TFC

Phenolic chemicals are secondary metabolites that plants produce under oxidative stress and are necessary to adapt to various adverse situations [1]. In the current investigation, TPC was measured using the Folin–Ciocalteu reagent, and it was discovered that the TPC ranged from 5.41 to 23.92 mgGAE/g (Table 1). According to earlier research, the total phenol content of Ajwa fruit ranged from 2.45 to 4.55 mgGAE/g. In contrast, this study found that URADP had a more significant percentage of total phenolic compounds [30,31]. Numerous studies have shown that the extraction solvent is crucial in the extraction of phenolic compounds. Compared to alcoholic extracts, the contents in hydroalcoholic extracts are always higher [32]. In addition, Eid et al. [33] stated that the phenolic content in Ajwa dates is also varied according to the ripening stage. Unripe Ajwa dates contain higher amounts of phenolic content than ripe fruits. Our experimental results also support this statement. In addition, flavonoids are the most abundant polyphenolic compounds found in Ajwa dates with pervasive dispersal. These polyphenolic compounds are mainly present within fruit skins in high concentrations with immense health benefits such as antioxidant and free radical scavenging activities [31,33]. In URADP extracts, TFC ranged from 6.81 to 24.20 mgCAE/g, which also agrees with the previous work [34].

As shown in Table 2, the linear effects of ethanol concentration (X_1_), extraction temperature (X_3_), quadratic component of (X_1_^2^), (X_2_^2^), and (X_3_^2^) and interaction of (X_1_X_2_), (X_1_X_3_) and (X_2_X_3_) exhibited significant effects on both TPC and TFC, except for the interaction of (X_2_X_3_), which has no significant effect on TPC. In addition, the regression coefficient (β) values verified the effect of extraction parameter on both TPC and TFC in the following order: TPC: X_1_^2^ > X_2_^2^ > X_3_ > X_3_^2^ > X_1_X_3_ > X_1_X_2_ > X_1_ and TFC: X_2_^2^ > X_1_^2^ > X_3_^2^ > X_3_ > X_2_X_3_ > X_1_X_2_ ≅ X_1_X_3_ > X_1_ (Table 2). The following second-order polynomial equations shown in Equations (1) and (2) demonstrate the relationships among TPC, TFC and their variables.

(1) $$TPC\left(Y_{1}\right)=23.39-0.3838X_{1}+0.0612X_{2}+3.06X_{3}-4.37X_{1}^{2}-4.10X_{2}^{2}\;\;\;\;\;\;\;-1.98X_{3}^{2}-0.5475X_{1}X_{2}-1.61X_{1}X_{3}+0.0825X_{2}X_{3}$$

(2) $$TFC\left(Y_{2}\right)=23.10+0.9656X_{1}-0.5854X_{2}+1.71X_{3}-3.78X_{1}^{2}-4.29X_{2}^{2}\;\;\;\;\;-2.81X_{3}^{2}-1.22X_{1}X_{2}-1.22X_{1}X_{3}+1.57X_{2}X_{3}$$

Three-dimensional response surface plots (Figure 1A,B) were constructed based on Equations (1) and (2), respectively, and were applied to clarify the interactive effects of the three variables on the TPC and TFC of URADP, respectively. The ethanol concentration (X_1_), extraction time (X_2_) and extraction temperature (X_3_) showed an interactive effect on both TPC and TFC, which increased readily with increasing ethanol concentration up to 60%, extraction time up to 90 min and extraction temperature up to 65 °C, followed by a decrease (Figure 1A,B). This could be because a medium concentration of ethanol may make the solvent more polar and dissolve more polyphenols, both polar and moderately polar ones [4]. Experiments in a previous comparative study revealed that the extraction of polyphenols from green tea leaves using a high hydrostatic pressure procedure augmented with the percentage of ethanol in the solvent; peaked at 50% ethanol and dropped after that [35]. Hence, the extraction of polyphenols in hydroalcoholic solution is highly efficient, as the polyphenols are highly soluble in these solutions. Furthermore, when ethanol is present at a moderate quantity in water, it can disrupt and break the architecture and structure of phospholipids that make up the lipid bilayer of membranes, affecting the penetrability of plant cells and thereby allowing for better extraction and diffusion of the polyphenolic compounds [36].

### 2.3. Model Validation

**Figure 2:**
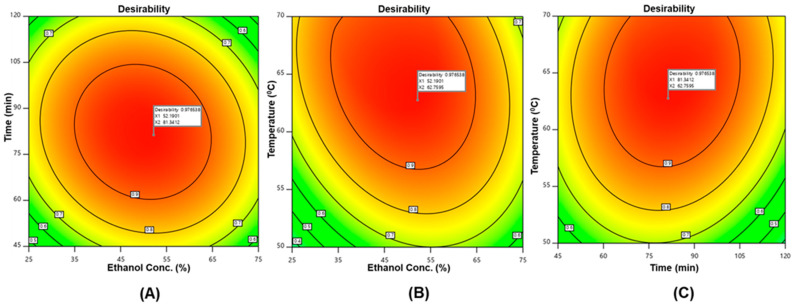
Desirability surface plot: as a function of (**A**) ethanol concentration and extraction time; (**B**) ethanol concentration and extraction temperature; (**C**) extraction time and temperature.

The parameters were forecasted using Derringer’s desirability function, allowing for the multivariate analysis to discover the ideal level for all responses in a single extraction [37]. Figure 2 shows the contour plot as a function of ethanol concentration, extraction time and temperature. In this study, the following conditions, (X_1_, 52%), (X_2_: 81 min), and (X_3_, 63 °C), were used to achieve the maximal overall desirability D = 0.977. Under these optimal conditions, the predicted values for TPC and TFC are 23.98 mgGAE/g and 23.39 mgCAE/g, respectively. To verify the sufficiency of the model equations, a triplicate experiment was conducted in the optimal conditions predicted by Derringer’s desire model and it found the TPC and TFC values to be 24.20 ± 0.096 mgGAE/g and 22.92 ± 1.19 mgCAE/g, respectively. As stated in Table 3, the relative standard deviations (RSDs) of TPC and TFC showed that the predicted values for all groups were very similar to the experimental results. This result is also supported by a prior report [38]. The suitability of the response surface methodology model for quantitative predictions was verified by a satisfactory agreement between the predicted and measured values.

### 2.4. Comparison of Optimized Extraction Condition with Other Extraction Methods Using Different Solvents

**Figure 3:**
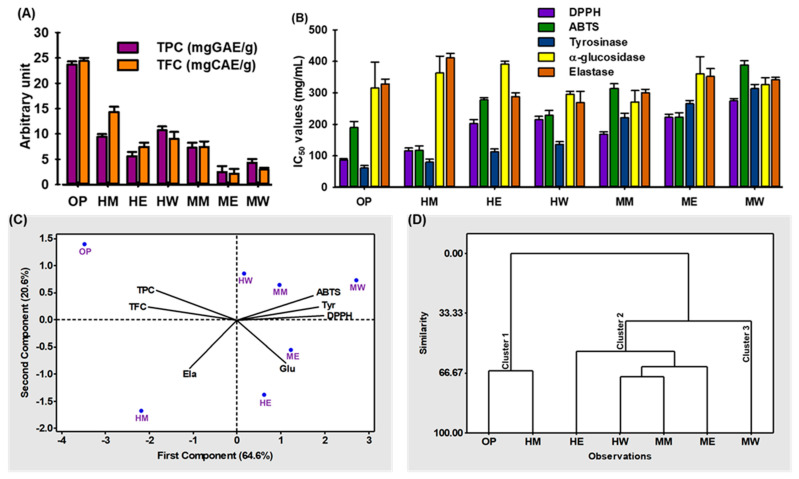
The pharmacological activities of the different extracts of URADP. (**A**) Total phenolic content (TPC) and total flavonoid content (TFC). (**B**) IC_50_ values of DPPH–, ABTS– radical scavenging, tyrosinase, α-glucosidase and elastase enzyme inhibition activity. (**C**) Biplot (scores of samples and load factors of each variable) of the principal component analysis (PCA). (**D**) Hierarchical cluster analysis (HCA). OP: heat extract with optimized condition, HM: heat extract with 100% methanol, HE: heat extract with 100% ethanol, HW: heat extract with 100% H_2_O, MM: maceration extract with 100% methanol, ME: maceration extract with 100% ethanol, MW: maceration extract with 100% H_2_O.

To demonstrate the effectiveness of the optimized method in extracting TPC and TFC, a comparative study was performed. As shown in Figure 3A, higher yields of TPC and TFC were obtained using hydroalcoholic solvent in heat extraction instead of methanol, ethanol and water for heat and maceration extraction. The extraction efficiency of TPC and TFC of different solvents and conditions are presented as heat extract with optimized condition (OP) > heat extract with 100% H_2_O (HW) > heat extract with 100% methanol (HM) > maceration extract with 100% methanol (MM) > heat extract with 100% ethanol (HE) > maceration extract with 100% H_2_O (MW) > maceration extract with 100% ethanol (ME) and OP > HM > HW > MM > HE > MW > ME, respectively. This result indicated that hydroalcoholic solvent with heat extraction was more efficient than that of other solvents with both heat and maceration techniques. The results also coincided with those obtained for the extraction of TPC and TFC from dates [30,31,32].

In addition, the pharmacological properties, such as antioxidant, tyrosinase, α-glucosidase, and elastase enzyme inhibitory activities, of various URADP extracts were intensively examined to determine their potential for application. Antioxidant components often have a potent ability to scavenge free radicals, preserving DNA and proteins from damage. Therefore, antioxidant chemicals have been utilized to treat a variety of diseases. DPPH^•^ and ABTS^•+^ has been frequently used as a representative reagent for examining the free radical scavenging activities of bioactive compounds. To quantify the antioxidant activities of different extracts/compounds, the concentration of the samples required to scavenge 50% of radicals (IC_50_) was measured. A smaller IC_50_ value indicates an increase in free-radical scavenging ability [38].

As anticipated, OP showed the lowest IC_50_ values (87.56 ± 1.21 mg/mL) for DPPH-, whereas HM had the lowest IC_50_ values (105.56 ± 0.98 mg/mL) for ABTS-radical scavenging activity. In addition, OP had the lowest IC_50_ values of 59.53 ± 1.02 mg/mL and 221.59 ± 2.52 mg/mL for tyrosinase and α-glucosidase enzyme inhibition, respectively. In contrast, the IC_50_ values (299.05 ± 2.52 mg/mL) for elastase enzyme inhibition were achieved by HW. To calculate the correlation between phenols, flavonoids, antioxidant and enzymes inhibition activity of different enriched products, the Pearson coefficient (ρ) method (supplementary data Table S2) was assessed. A negative ρ value (−1) represents the perfect positive correlation between polyphenols, free radical scavenging and enzyme inhibition ability using IC_50_. The results revealed very strong correlations for DPPH-radical scavenging and tyrosinase inhibition activity (*p* < 0.01) with TFC and (*p* < 0.05) for TPC. In contrast, there was no strong correlation shown between polyphenolic content with ABTS–radical scavenging, α–glucosidase and elastase enzyme inhibition activity. These data are in accordance with other studies that show that higher phenol content augments the antioxidant activity [39,40].

### 2.5. Chemometric Analysis

Chemometric analysis is the process of better understanding chemical information using mathematical and statistical methods. It is also the process of correlating quality characteristics to analytical instrument data. It has been used to investigate the relationship between antioxidant components and the antioxidant potentiality of various plant extracts [41]. This study used two chemometric techniques—principal component analysis (PCA) and hierarchical cluster analysis (HCA)—to find how the extraction method affected TPC, TFC, antioxidant effects, and other enzyme-inhibitory activities of URADP. PCA analysis reduces the dimensions of the data set and analyzes the responses based on the correlation between data samples. PCA could also find the variable that makes the most difference in the data set [41]. The loading plots were used to determine correlations between the study’s variables. The antioxidant activity, TPC, TFC, and other enzyme inhibitory activities were all included in these loading plots (Figure 3C). A total of 64.6% of the data set’s variability was accounted for by the first principal component (PC1), which also had the highest eigenvalue of 4.52. Meanwhile, 20.6% of the variability was represented by the PC2, which had an eigenvalue of 1.44. According to Figure 3C, the TPC and TFC, which point in the opposite direction from the IC_50_ loading vectors, may have the most significant potential to contribute to DPPH–, ABTS–radical scavenging, and tyrosinase inhibitory capacities. According to Pearson’s correlation analysis, the TPC and TFC were strongly linked with the antioxidant and tyrosine kinase inhibitory actions, supporting the PCA result (supplementary data Table S2). However, neither TPC nor TFC substantially impacted the activities of elastase and α–glucosidase. Additionally, all variables resulting from comparing the first two PCs (Figure 3C) revealed the existence of three different extract sample groups. Due to their high bioactive component concentration, antioxidant, and tyrosinase inhibitory activity, OP and HM made up Cluster I. In contrast, HE, HW, MM, and ME were made up of Cluster II since they had a mixed record regarding bioactive chemicals, antioxidant activity, and enzyme inhibition. Due to its inferior performance in TPC, TFC, antioxidant, and enzyme inhibition potentiality, extract MW made up Cluster III. Based on similarities, HCA was used to classify distinct solvent-based extraction techniques under research (Figure 3D).

### 2.6. Secondary Metabolites Profiling in URADP by High-Resolution Mass Spectrometry

Secondary metabolites in the URADP extracts were identified using ESI-MS/MS in the negative ionization modes. As indicated in Table 4, seventy (70) compounds were identified in the negative mode using MS^n^ data from the mass of the precursor ion, fragments, recognized fragmentation patterns for the given classes of compounds, and neutral mass loss, and from comparisons with the existing literature and searches in online databases. Furthermore, the significance of these results was determined by finding the confidence level. Level 3 denotes a tentative candidate, whereas level 2 indicates the probable structure of the identified compound [42].

#### 2.6.1. Phenolic Acids

**Figure 4:**
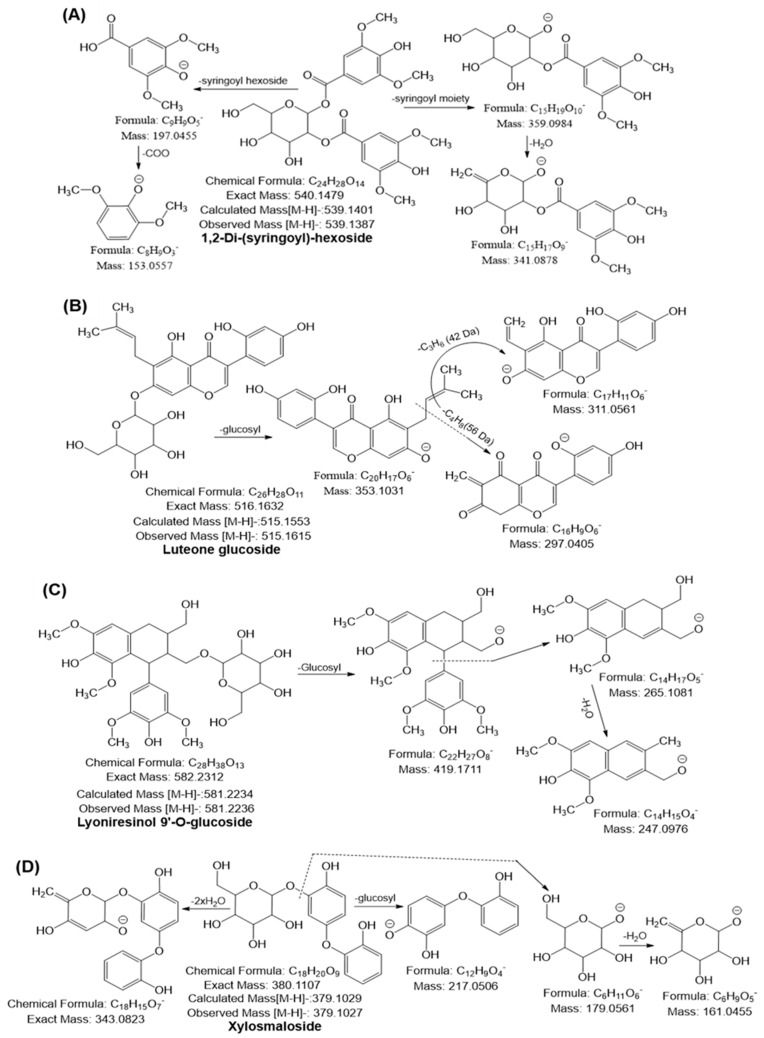
Tentative mass fragmentation behavior of 1,2-Di-(syringoyl)-hexoside (**A**), luteone glucoside (**B**), lyoniresinol 9-*O*-glucoside (**C**) and xylosmaloside (**D**).

A phenolic acid may lose its methyl (15 Da), hydroxyl (18 Da), or carboxyl (44 Da) moiety to form a specific fragment ion [42,43]. The fragmentation of a phenolic acid glycoside begins with the cleavage of the glycosidic link to yield the *m/z* of the phenolic acid and the corresponding loss of the sugar molecule (neutral mass loss of 162 Da). Thus, compounds 1–8 were tentatively identified as hydroxybenzoylhexose, coumaroylshikimic acid, vanillic acid glucoside, caffeoylshikimic acid, quinic acid hexoside, 5-feruloylquinic acid, caffeic acid derivatives, sinapic acid hexoside, caffeoyl shikimic acid hexoside, caffeoyl shikimic acid hexoside, and quinic acid derivatives, respectively [44,45]. Previous studies stated that p-coumaric acid, gallic acid and ferulic acid derivatives were the most dominant phenolic compounds in Ajwa dates [33]. In addition, compound 9 was tentatively identified as 1,2-di-(syringoyl)-hexoside with molecular formula (C_24_H_28_O_14_), which yielded a deprotonated ion [M–H]^—^ at *m/z* 539.1377 and generated the following fragment ions: *m/z* at 359.09 ([M–H–syringoyl moiety]), 341.08 ([M–H–syringoyl moiety–H_2_O]), 197.04 (syringic acid), and 153.05 because of the loss of a water molecule from ion *m/z* 197.04 (Figure 4A). Compound 9 has been identified for the first time in URADP.

#### 2.6.2. Flavonoids

Numerous studies demonstrated that each subgroup of flavonoids exhibits a different fragmentation behavior in MS^2^ analysis. The cleavage of the C-ring bonds (retro-Diels-Alder, i.e., RDA mechanism) produces ions with the A– or B–ring and some part of the C–ring, which is the most common fragmentation of flavonoids, and notable losses of small neutral molecules, such as CO (28 Da), C_2_H_2_O (42 Da), COO (44 Da), and 2CO (56 Da). [5,42,43]. Based on a comparison of the fragmentation patterns with those previously published in the literature, compounds 10-15 were identified as luteolin, catechin or epicatechin, chrysoeriol, quercetin, epigallocatechin, and methoxysinensetin, respectively [5,42,43,44,45]. Flavonoids are frequently glycosylated. The glycoside residues can be linked to the *O* and *C* atoms of the flavonoids, resulting in *O*-glycosides, *C*-glycosides, and *O-C*-glycosides. The typical fragmentation of *O*-glycosides produces neutral species corresponding to sugar units (hexoses, 162 Da; deoxyhexoses, 146 Da; pentoses, 132 Da) and an aglycone ion. Conversely, *C*-glucosides produce a sequence of fragments because of the cleavage of the C–C bonds with the sugar moiety; examples of such fragments are [M–H–60]^—^, [M–H–90]^—^, and [M–H–120]^—^, which serve as the hallmark diagnostic ions of glycone. Furthermore, in the case of *O-C* mixed glycosides, the cleavage of the *O*-glycosidic link is frequently observed in the first step [46,47]. Compounds 17, 19, 21–25, 27–30, and 32–40 were identified as naringenin rhamnoside, biochanin A 7-glucoside, afrormosin 7-glucoside, chrysoeriol hexoside, isoquercitrin, epicatechin 4’-glucuronide, isorhamnetin hexoside, luteolin hexosyl sulfate, chrysoeriol hexosyl sulfate, isoquercitrin sulfate, procyanidin B2, luteolin rhamnosyl hexoside, chrysoeriol rhamnosyl hexoside, isorhamnetin rhamnosyl hexoside, isorhamnetin diglucoside quercetin 3-O-rhamnoside 7-O-glucoside, isorhamnetin 3-O-rhamnosyl glucoside, quercetin xylosyl rutinoside, luteolin rhamnosyl dihexoside, quercetin glucosyl-rutinoside, Isorhamnetin rhamnosyl dihexoside and epicatechin-(2α→7,4α→8)-epicatechin glucoside, respectively, based on the similarities observed in the comparison of their fragmentation behaviors and with the behaviors reported in the literature [5,42,43]. The deprotonated molecular ion [M–H]^—^ at *m/z* 515.1611 exhibited MS^2^ fragment ions at *m/z* 353.10 by loss of glucosyl (162 Da). The ion at *m/z* 353.10 further yielded the MS^3^ ion at *m/z* 311 and 297.04 through the loss of 42 and 56 Da. Thus, compound 26 was tentatively identified as luteone glucoside, which has been identified for the first time in URADP (Figure 4B). Moreover, the monoisotopic mass [M–H]^—^ at *m/z* 581.2236 yielded a characteristic fragment ion at *m/z* 419.17 by loss of hexosyl moiety (162 Da), *m/z* at 265.10 and 247.09 by cleavage between the α- and β-position, followed by loss of H_2_O confirming the presence of lyoniresinol. It has been also identified for the first time in URADP (Figure 4C). Mounting evidence revealed that the Ajwa date fruit is enriched with active flavonoids and flavonoid glycosides (mainly as *O*-glycosides), which depend on the different ripening stages, and where significant quantities of quercetin, naringenin, apigenin, luteolin and kaempferol were found using LC-MS/MS techniques [30,31,32,44,45]. Furthermore, hydrolyzable tannins (HTs) are a broad category of polyphenolic compounds found in plants. During mass spectroscopy fragmentation, HTs frequently exhibit neutral losses of galloyl (152 Da). Compounds 18 and 20 have been characterized as epicatechin-3-gallate and epicatechin-3-(3-methylgallate), respectively, based on the MS and MS^2^ data and previously cited literature and were first identified in URADP [44].

#### 2.6.3. Sugar Molecules

Further, compound 49 was tentatively identified as xylosmaloside with molecular formula C_18_H_20_O_9_, and this compound generated the deprotonated ion [M–H]^—^ at *m/z* 379.1027 and the following mass fragmentation pattern: *m/z* 343.08 ([M–H–36 Da]), 217.05 ([M–H–162 Da]), 179.05 (xylose) and 161.04 ([M–H–179.05–18 Da]) (Figure 4D). This compound was also identified for the first time in RADP. Compounds 41–48 were confirmed as sugar molecules from comparison of their deprotonated ion mass and fragmentation behaviors with those reported in the literature and online databases [42,43,48,49,50].

#### 2.6.4. Carboxylic Acids and Fatty Acids

From comparisons of the mass and the fragmentation behaviors of the precursor ion based on mass spectroscopic analysis reported in the literature and various online databases [42,43,48,49,50], compounds 50–70 were identified as carboxylic acids and fatty acids (Table 4).

## 3. Materials and Methods

### 3.1. Sample Collection and Preparation

A scientific officer at the National Herbarium and Genebank of Saudi Arabia recognized unripe Ajwa date fruits (voucher specimen No. NHG005) obtained from an Ajwa date farm in Al-Madina Al-Munawara, Saudi Arabia, and they were kept in our lab for additional research. Unripe Ajwa date pulp (URADP) was separated, dried outside, chopped into small pieces, and ground in a sterilized laboratory blender (model 7011HS, Osaka Co. Ltd., Kita-Ku, Osaka, Japan). The powdered samples were maintained in an airtight container covered in aluminum foil and chilled before extraction.

### 3.2. Extraction Methods

Two distinct techniques and three different solvents (ethanol, methanol, and distilled water) were used for solvent extractions. The maceration method was primarily chosen because it is straightforward and inexpensive. In contrast, heat extraction was carried out in anticipation of a shorter extraction time since temperature may aid in breaking the plant cell wall of an empty palm fruit during heat extraction.

As stated by Mollica et al. [51], the maceration process was carried out with continuous stirring. Briefly, the plant materials (10 g) were soaked in 200 mL of the solvents, and extractions were performed with stirring at 250 rpm for 24 h at room temperature. Choi et al. [5] stated that 10 g of the extract and 200 mL of the solvents were used for heat extraction, which was carried out at 60 °C for 1 h. Following the extraction process, each extract was filtered using Whatman no. 1 filter paper (Schleicher & Schuell, Keene, NH, USA). The solvents were then removed using a rotary evaporator (Tokyo Rikakikai Co. Ltd., Tokyo, Japan) at 50 °C and 50 rpm. Finally, the extracts were lyophilized using a freeze dryer (Il-shin Biobase, Goyang, Republic of Korea). Before further research, the URADP extract was kept at −20 °C.

### 3.3. Total Phenolic Content (TPC) and Total Flavonoid Content (TFC)

The total phenolic content (TPC) and total flavonoid content (TFC) in URADP extracts were determined by the Folin–Ciocalteu test and the aluminum chloride colorimetric method, respectively [39]. The TPC (y = 0.0512x + 0.0018; r2 = 0.9835) and TFC (y = 0.014x + 0.0021; r2 = 0.9994) were determined using the corresponding regression equations for the calibration curves. The TPC was expressed in terms of the gallic acid equivalent (mg)/dry weight sample (g) and the TFC in terms of the catechin equivalent (mg)/dry weight sample (g).

### 3.4. Antioxidant Assay and Enzyme Inhibitory Effects

The antioxidant and enzyme inhibitory capability of various URADP extracts was evaluated using the procedures outlined in earlier publications [8,39,52,53]. Antioxidant experiments employed ascorbic acid as a positive control. In contrast, specific enzyme inhibitors, including arbutin, acarbose, and epigallocatechin gallate (EGCG), were utilized for the mushroom tyrosinase, α-glucosidase, and elastase enzyme assays, respectively. The percentage inhibition of DPPH- and ABTS-scavenging, mushroom tyrosinase, α-glucosidase, and elastase activity was calculated using Equation (3).

(3) $$\left(\%\;inhibition\right)=\left[\left(1-\frac{Abs_{sample}}{Abs_{control}}\right)\right]\times \;100$$

where Abs_control_ and Abs_sample_ are the absorbance of the control and absorbance of the sample, respectively. Each sample was examined three times. Each sample’s 50% inhibitory concentration (IC_50_) value was also computed to compare various extraction method efficacies.

### 3.5. Experimental Design of RSM for the Extraction Process

The hot extraction method was used to optimize the extraction procedure of polyphenolic compounds from URADP. The RSM model was designed to extract phenolic chemicals from URADP using ethanol concentration (X_1_), extraction duration (X_2_), and temperature (X_3_) as independent process factors. Respondent factors included TPC and TFC (Y_1_ and Y_2_, respectively). A three-component, five-layer CCD was employed for the extractions (supplementary data Table S1). The second-order polynomial model equation (Equation (4)) describes the link between independent factors and replies.

(4) $$Y=\beta_{0}+\overset{n}{\underset{i=1}{\sum }}\beta_{i}X_{i}+\overset{n}{\underset{i=1}{\sum }}\beta_{ii}X_{ii}^{2}+\overset{n-1}{\underset{i}{\sum }}\overset{n}{\underset{j}{\sum }}\beta_{ij}X_{ij}$$

where Y is the response variable and X_i_ and X_j_ are the independent coded variables; *β*_0_ denotes the constant coefficient, and *β_i_, β_ii_*, and *β_ij_* denote the coefficients of linear, quadratic, and interaction effects, respectively. Design Expert 11 was used for the RSM analysis and multiple linear regression (Stat-Ease, Minneapolis, Minnesota, USA). The model’s adequacy was tested using the determination coefficient (R^2^), the adjusted determination coefficient (Adj.R^2^), and the lack of fit test. The F value with *p* < 0.05 indicated statistical significance. The interaction outcome of each factor on the response value was represented in three-dimensional (3D) surface plots.

### 3.6. Optimal Extraction Condition and Validation of the Model

Derringer’s desire function was used to find the ideal conditions for maximizing all replies in a single experiment. Each response is turned into a unique desirability function ranging from 0 to 1 during this procedure. The component functions are then combined to create a total desirability function. The total desirability function is constructed using the following equation [4].

(5) $$D={\left(d_{1}^{w1}d_{2}^{w2}\ldots .d_{n}^{wn}\right)}^{1/\sum wi}$$

Response surface and desirability function analyses were used to determine the optimal extraction parameters. A triple experiment was carried out under ideal conditions, and the average experimental results were compared to the predicted results to verify the validity of the existing model. In addition, the experimental data were contrasted with the values that the model anticipated. Equation (5) was used to determine the relative standard deviation (RSD) and to compare the experimental and projected results.

(6) $$RSD\;\left(\%\right)=\frac{Standard\;deviation\;between\;predicted\;and\;experimental\;values}{Mean\;values\;between\;predicted\;and\;experimental\;values}\times \;100$$

The resulting data were analyzed and optimized for all response circumstances when the RSD% was <10. Additionally, the electrospray ionization mass spectrometry (ESI-MS)/MS profiles of phenolic compounds were found under ideal circumstances.

### 3.7. Analysis of Chemical Compounds by ESI-MS/MS

The Q-Exactive Orbitrap mass spectrometer (Thermo Fisher Scientific INC., San Jose, CA, USA) was used to conduct the negative (−) mode ESI-MS investigations. A 500 μL graded syringe (Hamilton Company Inc., Reno, NV, USA) and a 15 μL/min syringe pump (Model 11, Harvard, Holliston, MA, USA) were used to immerse the sample in the ESI source. The normal negative mode ESI-MS conditions were as follows: mass resolution of 140,000 (full width at half maximum, FWHM), sheath gas flow rate of 5, seep gas flow rate of 0, auxiliary gas flow rate of 0, spray voltage of 4.20 kV, capillary temperature of 320 °C, S-lens Rf level, and automatic gain control of 5 × 10^6^. The MS/MS studies were performed using the same instrument using three distinct stepwise normalized collision energies (10, 30, and 40) [5]. The Xcalibur 3.1 with Foundation 3.1 (Thermo Fisher Scientific Inc. Rockford, IL, USA) was used to process the collected mass spectral data. The *m/z* peaks were tentatively identified by comparing their calculated (exact) masses of deprotonated (M–H) adducts with the *m/z* values and ESI-MS/MS fragmentation patterns from the in-house MS/MS database and online databases such as FooDB [49], METLIN [50], CFM-ID 4.0 [48]. The chemical structure was drawn using ChemDraw Professional 15.0 (PerkinElmer, Waltham, MA, USA).

### 3.8. Statistical Analysis

All data were reported as the mean ± standard deviation of at least three independent experiments (n = 3), each with three sample replicates. One-way analysis of variance (ANOVA), followed by Dunnett’s multiple comparison test, was executed using SigmaPlot Version 12.5 (Systat Software, Inc., Chicago, IL, USA) to determine statistical significance at *p* < 0.001, *p* < 0.01, and *p* < 0.05. Principle component analysis (PCA) was performed to analyze the effect of the extraction method on TPC, TFC, antioxidant, mushroom tyrosinase, α-glucosidase and elastase enzyme inhibition and to learn the correlations between these variables. PCA was carried out using Minitab Statistical Software (Version 18.0, Minitab Inc., Enterprise Drive State College, PA, USA).

## 4. Conclusions

This study was the first investigation on optimizing the solvent extraction conditions on URADP using RSM, and high-resolution mass spectroscopic analysis revealed the presence of phenolic acids, flavonoids, lignans, etc. Optimal conditions (52% ethanol, extraction time of 81 min, and extraction temperature of 63 °C) were determined. Under these conditions, the maximum TPC and TFC were obtained as 24.25 mgGAE/g and 23.98 mgCAE/g, respectively. Optimized extract (OP) and heat extract made using 100% methanol (HM) also showed significant antioxidant and anti-tyrosinase enzyme activity compared to other extracts. Furthermore, on the basis of their bioactive components and biological activities, chemometric analysis showed a substantial association between the HM and OP by grouping them together. However, the mechanism underlying URADP’s antioxidant and depigmenting actions is still unknown. The antioxidant and depigmenting actions of URADP are still being confirmed in investigations using cells and animal models. Based on these outcomes, we can conclude that these findings can be used as the basis for a broad commercial application of URADP, a promising candidate for an antioxidant and tyrosinase as enzymatic inhibition functional food, in nutraceutical food and pharmaceutical industries.

## Figures and Tables

**Table 1:** Central composite design (CCD) for independent variables and corresponding response values (experimental).

Run	Independent Variables			Responses			
	(X_1_)	(X_2_)	(X_3_)	TPC (Y_1_)		TFC (Y_2_)	
				Exp.	Pred.	Exp.	Pred.
1	100	82.5	60	5.41 ± 0.28	5.70	11.02 ± 0.33	9.90
2	50	82.5	60	23.69 ± 0.43	23.34	21.05 ± 0.62	23.10
3	75	120	70	13.75 ± 0.54	14.17	12.9 ± 0.15	13.44
4	50	15	60	10.24 ± 0.76	9.75	11.52 ± 0.25	10.24
5	75	120	50	10.21 ± 0.61	10.60	7.83 ± 0.39	9.31
6	50	82.5	60	23.12 ± 0.12	23.34	24.20 ± 0.20	23.10
7	0	82.5	60	6.53 ± 0.12	6.73	6.85 ± 0.16	6.04
8	25	45	70	17.50 ± 0.69	17.62	11.52 ± 0.46	11.97
9	25	120	50	8.88 ± 0.45	8.99	6.81 ± 0.35	7.39
10	50	82.5	80	23.00 ± 0.43	22.89	16.05 ± 0.75	15.28
11	50	82.5	60	23.10 ± 0.72	23.34	23.59 ± 0.36	23.10
12	50	82.5	60	23.51 ± 0.16	23.34	24.01 ± 0.43	23.10
13	50	82.5	60	23.92 ± 0.54	23.34	24.01 ± 0.63	23.10
14	25	45	50	7.85 ± 0.72	7.94	7.85 ± 0.55	9.25
15	50	150	60	10.11 ± 0.46	9.97	9.25 ± 0.25	8.14
16	75	45	50	11.01 ± 0.04	11.74	15.25 ± 0.80	16.05
17	25	120	70	19.22 ± 0.58	19.00	15.25 ± 0.92	16.38
18	50	82.5	40	10.05 ± 0.18	9.64	9.59 ± 0.22	8.43
19	50	82.5	60	23.10 ± 0.53	23.34	23.25 ± 0.59	23.10
20	75	45	70	14.58 ± 0.54	14.98	12.56 ± 0.27	13.92

X_1_: ethanol concentration (%); X_2_: time (min); X_3_: temperature (°C); TPC: total phenolic content (mg gallic acid equivalent/g dry weight extract); TFC: total flavonoid content (mg catechin equivalent/g dry weight extract). Exp.: experimental value; Pred.: predicted value.

**Table 2:** ANOVA for quadratic model.

ANOVA for Quadratic Model for TPC							
Source	RC	SS	DF	MS	F Value	*p* Value	
Model		843.91	9	93.77	347.63	<0.0001	Significant
Intercept	23.39						
Linear terms							
X_1_	−0.3838	2.36	1	2.36	8.74	0.0144	Significant
X_2_	0.0612	0.0542	1	0.0542	0.2010	0.6635	Nonsignificant
X_3_	3.06	150.31	1	150.31	557.25	<0.0001	Significant
Interaction terms							
X_1_X_2_	−0.5475	2.40	1	2.40	8.89	0.0138	Significant
X_1_X_3_	−1.61	20.74	1	20.74	76.88	<0.0001	Significant
X_2_X_3_	0.0825	0.0544	1	0.0544	0.2019	0.6628	Nonsignificant
Quadratic terms							
X_1_^2^	−4.37	484.62	1	484.62	1796.67	<0.0001	Significant
X_2_^2^	−4.10	292.30	1	292.30	1083.65	<0.0001	Significant
X_3_^2^	−1.98	99.41	1	99.41	368.54	<0.0001	Significant
Lack of Fit		2.07	5	0.4145	3.32	0.1071	Nonsignificant
Pure error		0.6247	5	0.1249			
R^2^							0.9968
Adjusted R^2^							0.9939
Adeq Precision							49.6969
C.V.%							3.39
**ANOVA for quadratic model for TFC**							
Model		751.10	9	83.46	36.64	<0.0001	Significant
Intercept	23.10						
Linear terms							
X_1_	0.9656	14.92	1	14.92	6.55	0.0284	Significant
X_2_	−0.5854	4.96	1	4.96	2.18	0.1708	Nonsignificant
X_3_	1.71	46.96	1	46.96	20.61	0.0011	Significant
Interaction terms							
X_1_X_2_	−1.22	11.93	1	11.93	5.24	0.0451	Significant
X_1_X_3_	−1.22	11.83	1	11.83	5.20	0.0458	Significant
X_2_X_3_	1.57	19.63	1	19.63	8.62	0.0149	Significant
Quadratic terms							
X_1_^2^	−3.78	363.27	1	363.27	159.47	<0.0001	Significant
X_2_^2^	−4.29	320.24	1	320.24	140.58	<0.0001	Significant
X_3_^2^	−2.81	200.73	1	200.73	88.12	<0.0001	Significant
Lack of Fit		15.83	5	3.17	2.28	0.1938	Nonsignificant
Pure error		6.95	5	1.39			
R^2^							0.9706
Adjusted R^2^							0.9441
Adeq Precision							15.9930
C.V.%							10.25

RC: regression coefficient; SS: sum of squares; MS: mean square.

**Table 3:** Experiment data of the validation of predicted values at optimal extraction conditions of URADP.

Response	Exp.	Pred.	Std	RSD (%)
TPC (mgGAE/g)	24.25 ± 1.02	23.97	0.20	0.82
TFC (mgCAE/g)	23.98 ± 0.65	23.39	0.42	1.76

Optimal condition: ethanol concentration (%): 51.97%; time (min): 81.38; temperature (°C): 62.76. Exp.: experimental value; Pred.: predicted value; Std: standard deviation; RSD: relative standard deviation.

**Table 4:** List of tentative identified compounds of the optimized extract of URADP by electrospray ionization mass spectrometry (ESI-MS)/MS.

Group	No.	Compound Name	EF	OM (*m/z*)^–^	CM (*m/z*)^–^	MS/MS (Negative Mode)	CE	CL
Phenolic acids and derivatives	1	4-Hydroxybenzoyl glucose	C_13_H_16_O_8_	299.0773	299.0766	137.02, 163.02	20	2
	2	Coumaroylshikimic acid	C_16_H_16_O_7_	319.0824	319.0817	173.04, 163.03, 145.02	20	2
	3	Vanillic acid glucoside	C_14_H_18_O_9_	329.0873	329.0872	167.03, 152.02, 123.04	20	2
	4	Caffeoylshikimic acid	C_16_H_16_O_8_	335.0776	335.0772	179.01, 161.03, 155.03, 137.05	20	2
	5	Quinic acid hexoside	C_13_H_22_O_11_	353.1085	353.1084	191.05, 173.04, 179.05	20	2
	6	5-Feruloylquinic acid	C_17_H_20_O_9_	367.1046	367.1029	191.08, 173.04, 127.01	30	2
	5	Caffeic acid derivatives	C_18_H_18_O_9_	377.0885	377.0878	341.10, 215.03, 179.06, 161.04, 135.05	10	2
	6	Sinapic acid hexoside	C_17_H_22_O_10_	385.1141	385.1135	223.06, 205.05	10	2
	7	Caffeoyl shikimic acid hexoside	C_22_H_26_O_13_	497.1297	497.1295	335.01, 178.02, 161.03, 155.03, 135.02	20	2
	8	Quinic acid derivatives	C_19_H_34_O_17_	533.1718	533.1718	341.10, 191.05	30	2
	9	1,2-di-(syringoyl)-hexoside ^#^	C_24_H_28_O_14_	539.1377	539.1401	359.09, 341.08, 197.04, 153.05	30	3
Flavonoids and derivatives	10	Luteolin	C_15_H_10_O_6_	285.0405	285.0399	267.05, 241.03, 151.00, 133.02	20	2
	11	Catechin/Epicatechin	C_15_H_14_O_6_	289.0718	289.0712	245.04, 205.05, 179, 151.04, 137.02	20	2
	12	Chrysoeriol	C_13_H_16_O_8_	299.0561	299.0555	285.03, 153.01, 135.03, 125.03	20	2
	13	Quercetin	C_15_H_10_O_7_	301.0354	301.0348	273.02, 229.05, 179.01, 151.01	20	2
	14	Epigallocatechin	C_15_H_14_O_7_	305.0644	305.0661	287.05, 137.02, 125.02	20	2
	15	Methoxysinensetin ^#^	C_21_H_22_O_8_	401.1299	401.1236	371.11, 339.08, 191.71	20	2
	16	Epicatechin hydroxybenzoate ^#^	C_22_H_18_O_8_	409.0924	409.0923	289.07, 271.06, 137.02, 119.01	30	2
	17	Naringenin rhamnoside	C_21_H_22_O_9_	417.1245	417.1186	271.06, 187.03, 151.00, 119.05	20	2
	18	Epicatechin-3-gallate	C_22_H_18_O_10_	441.081	441.0821	371.04, 273.02, 135.10, 169.02	30	2
	19	Biochanin A 7-glucoside ^#^	C_22_H_22_O_10_	445.1195	445.1135	283.06, 239.03, 211.04, 132.02	30	2
	20	Epicatechin 3-(-methylgallate) ^#^	C_23_H_20_O_10_	455.1015	455.0978	289.02, 183.05, 124.01	30	2
	21	Afrormosin 7-glucoside	C_23_H_24_O_10_	459.1354	459.1291	297.07, 281.04, 267.06	20	2
	22	Chrysoeriol hexoside	C_22_H_22_O_11_	461.1085	461.1083	299.07, 283.02, 269.06	20	2
	23	Isoquercitrin	C_21_H_20_O_12_	463.0878	463.0876	301.05, 268.01, 179.02, 151.01	20	2
	24	Epicatechin 4’-glucuronide^#^	C_21_H_22_O_12_	465.1036	465.1033	289.15, 151.10, 137.08, 123.10	20	2
	25	Isorhamnetin hexoside	C_22_H_22_O_12_	477.1035	477.1033	315.05, 300.01, 179.05, 151.02	20	2
	26	Luteone glucoside	C_26_H_28_O_11_	515.1611	515.1553	353.10, 311.05, 297.04	20	3
	27	Luteolin hexosyl sulfate	C_21_H_20_O_14_S	527.0491	527.0495	447.05, 285.01, 241.06	20	2
	28	Chrysoeriol hexosyl sulfate	C_22_H_22_O_14_S	541.0645	541.0652	299.05, 284.05, 241.02	20	2
	29	Isoquercitrin sulfate	C_21_H_20_O_15_S	543.0441	543.0444	463.05, 301.01, 179.02, 151.01	20	2
	30	Procyanidin B2 ^#^	C_30_H_26_O_12_	577.1347	577.1346	451.10, 407.07, 289.07, 287.05, 125.02	20	2
	31	Lyoniresinol 9-glucoside ^#^	C_28_H_37_O_13_	581.2236	581.2234	419.17, 265.10, 247.09	20	2
	32	Luteolin rhamnosyl hexoside	C_27_H_30_O_15_	593.1507	593.1506	447.09, 285.03, 153.01, 135.04	20	2
	33	Chrysoeriol rhamnosyl hexoside	C_28_H_32_O_15_	607.1669	607.1663	461.10, 299.05, 153.01, 149.05	20	2
	34	Isorhamnetin rhamnosyl hexoside	C_28_H_32_O_16_	623.1617	623.1612	477.10, 315.05, 299.05, 165.05	20	2
	35	Isorhamnetin diglucoside	C_28_H_32_O_17_	639.1563	639.1561	447.01, 315.01	20	2
	36	Quercetin xylosyl rutinoside ^#^	C_32_H_38_O_20_	741.1846	741.1878	609.14, 301.03	10	2
	37	Luteolin rhamnosyl dihexoside	C_33_H_40_O_20_	755.2046	755.2034	709.16, 593.10, 575.05, 285.01	20	2
	38	Quercetin glucosyl-rutinoside	C_33_H_40_O_21_	771.1981	771.1983	609.14, 591.05, 301.03, 153.02, 125.00	20	2
	39	Isorhamnetin rhamnosyl dihexoside	C_34_H_42_O_21_	785.211	785.214	623.16, 477.10, 315.05	20	2
	40	Epicatechin-(2α→7,4α→8)-epicatechin glucoside ^#^	C_36_H_34_O_17_	737.1721	737.1718	721.02, 577.05, 425.05, 195.02	30	2
Sugar molecules	41	Ribonic acid	C_5_H_10_O_6_	165.0421	165.0418	149.04, 105.01, 87.00, 75.00	10	2
	42	L-Galactose	C_6_H_12_O_6_	179.0572	179.0561	161.04, 143.03, 113.02, 101.02,	10	2
	43	Gluconic acid	C_6_H_12_O_7_	195.0522	195.0504	177.05, 159.02, 129.05, 98.90	10	2
	48	Sedoheptulose	C_7_H_14_O_7_	209.0679	209.068	191.05, 179.05, 149.04,	20	2
	49	Xylosmaloside ^#^	C_18_H_20_O_9_	379.1027	379.1029	343.08, 217.05, 179.05, 161.04	20	3
Carboxylic acids	50	Fumaric acid	C_4_H_4_O_4_	115.005	115.0037	71.01	10	2
	51	Glutaconic acid	C_5_H_6_O_4_	129.0203	129.0203	111.00, 85.02	10	2
	52	Glutaric acid	C_5_H_8_O_4_	131.0355	131.035	113.00, 87.02	10	2
	53	3-Methylglutaconic acid	C_6_H_8_O_4_	143.0367	143.0361	99.03	20	2
	54	Methyl glutaric acid	C_6_H_10_O_4_	145.0521	145.0506	127.02, 101.02	10	2
	55	2-Hydroxyglutaric acid	C_5_H_8_O_5_	147.0301	147.0299	129.01, 99.03	10	2
	56	Hydroxymethyl glutaric acid	C_6_H_10_O_5_	161.0459	161.0455	143.03, 117.05, 99.04	10	2
	58	Citric acid	C_6_H_8_O_7_	191.0197	191.0197	173.00, 129.01, 111.00	20	2
Fatty acids	59	Palmitic acid	C_16_H_32_O_2_	255.233	255.233	237.23, 211.24, 197.22	20	2
	60	Linolenic acid	C_18_H_30_O_2_	277.2165	277.2169	259.20, 233.22, 205.21, 179.25,	10	2
	61	α-Linoleic acid	C_18_H_32_O_2_	279.2331	279.2330	261.22	10	2
	62	Oleic acid	C_18_H_34_O_2_	281.2487	281.2486	263.25, 181.21, 127.25	10	2
	63	Hydroxy octadecatrienoic acid ^#^	C_18_H_30_O_3_	293.212	293.0216	275.22	20	3
	64	Hydroxy octadecadienoic acid	C_18_H_32_O_3_	295.2276	295.2273	277.23	20	2
	65	Hydroxy octadecenoic acid	C_18_H_34_O_3_	297.2433	297.2429	279.23	20	2
	66	Dihydroxy octadecadienoic acid	C_18_H_32_O_4_	311.2246	311.2239	293.22, 275.23	20	2
	67	Dihydroxy octadecenoic acid	C_18_H_34_O_4_	313.2381	313.2378	295.23, 277.25, 183.32	20	2
	68	Dihydroxy octadecanoic acid	C_18_H_36_O_4_	315.2538	315.2535	297.23, 279.25,	20	2
	69	Trihydroxy octadecadienoic acid	C_18_H_32_O_5_	327.2176	327.2171	309.23, 291.25, 273.23	20	2
	70	Trihydroxy octadecenoic acid	C_18_H_34_O_5_	329.2346	329.2333	311.25, 293.26, 275.23	20	2

EF: elemental formula; OM: observed mass; CM: calculated mass; CE: collision energy (eV); CL: confidence level; ^#^ First time identification in Ajwa date.

## Data Availability

Upon request, Authors will provide the data.

## Supplementary Materials

The following supporting information can be downloaded at: https://www.mdpi.com/article/10.3390/ijms24043396/s1.

## References

[1] Simić V.M., Rajković K.M., Stojičević S.S., Veličković D.T., Nikolić N.Č., Lazić M.L., Karabegović I.T. (2016). Optimization of microwave-assisted extraction of total polyphenolic compounds from chokeberries by response surface methodology and artificial neural network. Sep. Purif. Technol..

[2] Bochi V.C., Barcia M.T., Rodrigues D., Speroni C.S., Giusti M.M., Godoy H.T. (2014). Polyphenol extraction optimisation from Ceylon gooseberry (*Dovyalis hebecarpa*) pulp. Food Chem..

[3] Samaram S., Mirhosseini H., Tan C.P., Ghazali H.M., Bordbar S., Serjouie A. (2015). Optimisation of ultrasound-assisted extraction of oil from papaya seed by response surface methodology: Oil recovery, radical scavenging antioxidant activity, and oxidation stability. Food Chem..

[4] Sedraoui S., Badr A., Barba M.G.M., Doyen A., Tabka Z., Desjardins Y. (2020). Optimization of the Ultrahigh-Pressure–Assisted Extraction of Phenolic Compounds and Antioxidant Activity from Palm Dates (*Phoenix dactylifera* L.). Food Anal. Methods.

[5] Choi H.-J., Naznin M., Alam M.B., Javed A., Alshammari F.H., Kim S., Lee S.-H. (2022). Optimization of the extraction conditions of Nypa fruticans Wurmb. using response surface methodology and artificial neural network. Food Chem..

[6] Javed A., Naznin M., Alam M.B., Fanar A., Song B.-R., Kim S., Lee S.-H. (2022). Metabolite Profiling of Microwave-Assisted Sargassum fusiforme Extracts with Improved Antioxidant Activity Using Hybrid Response Surface Methodology and Artificial Neural Networking-Genetic Algorithm. Antioxidants.

[7] Hou M., Hu W., Wang A., Xiu Z., Shi Y., Hao K., Sun X., Cao D., Lu R., Sun J. (2019). Ultrasound-Assisted Extraction of Total Flavonoids from Pteris cretica L.: Process Optimization, HPLC Analysis, and Evaluation of Antioxidant Activity. Antioxidants.

[8] Alam M.B., Ahmed A., Motin M.A., Kim S., Lee S.H. (2018). Attenuation of melanogenesis by Nymphaea nouchali (Burm. f) flower extract through the regulation of cAMP/CREB/MAPKs/MITF and proteasomal degradation of tyrosinase. Sci. Rep..

[9] Kim J.H., Jang D.H., Lee K.W., Kim K.D., Shah A.B., Zhumanova K., Park K.H. (2020). Tyrosinase Inhibition and Kinetic Details of Puerol A Having But-2-Enolide Structure from Amorpha fruticosa. Molecules.

[10] Kim D.S., Cha S.B., Park M.C., Park S.A., Kim H.S., Woo W.H., Mun Y.J. (2017). Scopoletin Stimulates Melanogenesis via cAMP/PKA Pathway and Partially p38 Activation. Biol. Pharm. Bull..

[11] Shah A.B., Yoon S., Kim J.H., Zhumanova K., Ban Y.J., Lee K.W., Park K.H. (2020). Effectiveness of cyclohexyl functionality in ugonins from Helminthostachys zeylanica to PTP1B and α-glucosidase inhibitions. Int. J. Biol. Macromol..

[12] Jakimiuk K., Gesek J., Atanasov A.G., Tomczyk M. (2021). Flavonoids as inhibitors of human neutrophil elastase. J. Enzym. Inhib. Med. Chem..

[13] Alam S.R., Newby D.E., Henriksen P.A. (2012). Role of the endogenous elastase inhibitor, elafin, in cardiovascular injury: From epithelium to endothelium. Biochem. Pharm..

[14] Ban Y.J., Baiseitova A., Nafiah M.A., Kim J.Y., Park K.H. (2020). Human neutrophil elastase inhibitory dihydrobenzoxanthones and alkylated flavones from the Artocarpus elasticus root barks. Appl. Biol. Chem..

[15] von Nussbaum F., Li V.M. (2015). Neutrophil elastase inhibitors for the treatment of (cardio)pulmonary diseases: Into clinical testing with pre-adaptive pharmacophores. Bioorg. Med. Chem. Lett..

[16] Delgado-Rizo V., Martínez-Guzmán M.A., Iñiguez-Gutierrez L., García-Orozco A., Alvarado-Navarro A., Fafutis-Morris M. (2017). Neutrophil Extracellular Traps and Its Implications in Inflammation: An Overview. Front. Immunol..

[17] Yang H., Biermann M.H., Brauner J.M., Liu Y., Zhao Y., Herrmann M. (2016). New Insights into Neutrophil Extracellular Traps: Mechanisms of Formation and Role in Inflammation. Front. Immunol..

[18] Tabaraki R., Nateghi A. (2011). Optimization of ultrasonic-assisted extraction of natural antioxidants from rice bran using response surface methodology. Ultrason. Sonochem..

[19] Bezerra M.A., Santelli R.E., Oliveira E.P., Villar L.S., Escaleira L.A. (2008). Response surface methodology (RSM) as a tool for optimization in analytical chemistry. Talanta.

[20] Yasin B.R., El-Fawal H.A., Mousa S.A. (2015). Date (*Phoenix dactylifera*) Polyphenolics and Other Bioactive Compounds: A Traditional Islamic Remedy’s Potential in Prevention of Cell Damage, Cancer Therapeutics and Beyond. Int. J. Mol. Sci..

[21] Al-Yahya M., Raish M., AlSaid M.S., Ahmad A., Mothana R.A., Al-Sohaibani M., Al-Dosari M.S., Parvez M.K., Rafatullah S. (2016). ‘Ajwa’ dates (*Phoenix dactylifera* L.) extract ameliorates isoproterenol-induced cardiomyopathy through downregulation of oxidative, inflammatory and apoptotic molecules in rodent model. Phytomed. Int. J. Phytother. Phytopharm..

[22] Almatroodi S.A., Khan A.A., Aloliqi A.A., Ali Syed M., Rahmani A.H. (2022). Therapeutic Potential of Ajwa Dates (*Phoenix dactylifera*) Extract in Prevention of Benzo(a)pyrene-Induced Lung Injury through the Modulation of Oxidative Stress, Inflammation, and Cell Signalling Molecules. Appl. Sci..

[23] Abdelghffar E.A., Obaid W.A., Mohammedsaleh Z.M., Ouchari W., Eldahshan O.A., Sobeh M. (2022). Ajwa dates (*Phoenix dactylifera* L.) attenuate cisplatin-induced nephrotoxicity in rats via augmenting Nrf2, modulating NADPH oxidase-4 and mitigating inflammatory/apoptotic mediators. Biomed. Pharmacother. Biomed. Pharmacother..

[24] Al-Shahib W., Marshall R.J. (2003). The fruit of the date palm: Its possible use as the best food for the future?. Int. J. Food Sci. Nutr..

[25] Rahmani A.H., Aly S.M., Ali H., Babiker A.Y., Srikar S., Khan A.A. (2014). Therapeutic effects of date fruits (Phoenix dactylifera) in the prevention of diseases via modulation of anti-inflammatory, anti-oxidant and anti-tumour activity. Int. J. Clin. Exp. Med..

[26] Boulenouar N., Marouf A., Cheriti A. (2011). Antifungal activity and phytochemical screening of extracts from Phoenix dactylifera L. cultivars. Nat. Prod. Res..

[27] Almusallam I.A., Ahmed I.A.M., Babiker E.E., Al Juhaimi F.Y., Fadimu G.J., Osman M.A., Al Maiman S.A., Ghafoor K., Alqah H.A. (2021). Optimization of ultrasound-assisted extraction of bioactive properties from date palm (*Phoenix dactylifera* L.) spikelets using response surface methodology. LWT.

[28] Ghafoor K., Sarker M.Z.I., Al-Juhaimi F.Y., Babiker E.E., Alkaltham M.S., Almubarak A.K. (2022). Extraction and Evaluation of Bioactive Compounds from Date (*Phoenix dactylifera*) Seed Using Supercritical and Subcritical CO_2_ Techniques. Foods.

[29] Pourshoaib S.J., Ghatrami E.R., Shamekhi M.A. (2022). Comparing ultrasonic-and microwave-assisted methods for extraction of phenolic compounds from Kabkab date seed (*Phoenix dactylifera* L.) and stepwise regression analysis of extracts antioxidant activity. Sustain. Chem. Pharm..

[30] Al-Turki S., Shahba M.A., Stushnoff C. (2010). Diversity of antioxidant properties and phenolic content of date palm (*Phoenix dactylifera* L.) fruits as affected by cultivar and location. J. Food Agric. Environ..

[31] Mohamed S.A., Awad M.A., El-Dengawy E.-R.F.A., Abdel-Mageed H.M., El-Badry M.O., Salah H.A., Abdel-Aty A.M., Fahmy A.S. (2016). Total phenolic and flavonoid contents and antioxidant activities of sixteen commercial date cultivars grown in Saudi Arabia. RSC Adv..

[32] Hamad I., AbdElgawad H., Al Jaouni S., Zinta G., Asard H., Hassan S., Hegab M., Hagagy N., Selim S. (2015). Metabolic Analysis of Various Date Palm Fruit (*Phoenix dactylifera* L.) Cultivars from Saudi Arabia to Assess Their Nutritional Quality. Molecules.

[33] Eid N.M.S., Al-Awadi B., Vauzour D., Oruna-Concha M.J., Spencer J.P.E. (2013). Effect of Cultivar Type and Ripening on the Polyphenol Content of Date Palm Fruit. J. Agric. Food Chem..

[34] Hassan S.M.A., Aboonq M.S., Albadawi E.A., Aljehani Y., Abdel-Latif H.M., Mariah R.A., Shafik N.M., Soliman T.M., Abdel-Gawad A.R., Omran F.M. (2022). The Preventive and Therapeutic Effects of Ajwa Date Fruit Extract Against Acute Diclofenac Toxicity-Induced Colopathy: An Experimental Study. Drug Des. Devel..

[35] Xi J., Wang B. (2013). Optimization of Ultrahigh-Pressure Extraction of Polyphenolic Antioxidants from Green Tea by Response Surface Methodology. Food Bioprocess Technol..

[36] Gurtovenko A.A., Anwar J. (2009). Interaction of Ethanol with Biological Membranes: The Formation of Non-bilayer Structures within the Membrane Interior and their Significance. J. Phys. Chem. B.

[37] Derrien M., Badr A., Gosselin A., Desjardins Y., Angers P. (2017). Optimization of a green process for the extraction of lutein and chlorophyll from spinach by-products using response surface methodology (RSM). LWT-Food Sci. Technol..

[38] Xu S., Li X., Liu S., Tian P., Li D. (2022). Juniperus sabina L. as a Source of Podophyllotoxins: Extraction Optimization and Anticholinesterase Activities. Int. J. Mol. Sci..

[39] Alam M.B., Ju M.K., Lee S.H. (2017). DNA Protecting Activities of Nymphaea nouchali (Burm. f) Flower Extract Attenuate t-BHP-Induced Oxidative Stress Cell Death through Nrf2-Mediated Induction of Heme Oxygenase-1 Expression by Activating MAP-Kinases. Int. J. Mol. Sci..

[40] Rebollo-Hernanz M., Cañas S., Taladrid D., Segovia Á., Bartolomé B., Aguilera Y., Martín-Cabrejas M.A. (2021). Extraction of phenolic compounds from cocoa shell: Modeling using response surface methodology and artificial neural networks. Sep. Purif. Technol..

[41] Sunarwidhi A.L., Hernawan A., Frediansyah A., Widyastuti S., Martyasari N.W.R., Abidin A.S., Padmi H., Handayani E., Utami N.W.P., Maulana F.A. (2022). Multivariate Analysis Revealed Ultrasonic-Assisted Extraction Improves Anti-Melanoma Activity of Non-Flavonoid Compounds in Indonesian Brown Algae Ethanol Extract. Molecules.

[42] Naznin M., Badrul Alam M., Alam R., Islam S., Rakhmat S., Lee S.-H., Kim S. (2023). Metabolite profiling of Nymphaea rubra (Burm. f.) flower extracts using cyclic ion mobility–mass spectrometry and their associated biological activities. Food Chem..

[43] Alam M.B., Naznin M., Islam S., Alshammari F.H., Choi H.J., Song B.R., Kim S., Lee S.H. (2021). High Resolution Mass Spectroscopy-Based Secondary Metabolite Profiling of Nymphaea nouchali (Burm. f) Stem Attenuates Oxidative Stress via Regulation of MAPK/Nrf2/HO-1/ROS Pathway. Antioxidants.

[44] Najm O.A., Addnan F.H., Mohd-Manzor N.F., Elkadi M.A., Abdullah W.O., Ismail A., Mansur F.A.F. (2021). Identification of Phytochemicals of Phoenix dactylifera L. Cv Ajwa with UHPLC-ESI-QTOF-MS/MS. Int. J. Fruit Sci..

[45] Nematallah K.A., Ayoub N.A., Abdelsattar E., Meselhy M.R., Elmazar M.M., El-Khatib A.H., Linscheid M.W., Hathout R.M., Godugu K., Adel A. (2018). Polyphenols LC-MS2 profile of Ajwa date fruit (*Phoenix dactylifera* L.) and their microemulsion: Potential impact on hepatic fibrosis. J. Funct. Foods.

[46] Kachlicki P., Piasecka A., Stobiecki M., Marczak Ł. (2016). Structural Characterization of Flavonoid Glycoconjugates and Their Derivatives with Mass Spectrometric Techniques. Molecules.

[47] Vukics V., Guttman A. (2010). Structural characterization of flavonoid glycosides by multi-stage mass spectrometry. Mass Spectrom. Rev..

[48] Wang F., Liigand J., Tian S., Arndt D., Greiner R., Wishart D.S. (2021). CFM-ID 4.0: More Accurate ESI-MS/MS Spectral Prediction and Compound Identification. Anal. Chem..

[49] Naveja J.J., Rico-Hidalgo M.P., Medina-Franco J.L. (2018). Analysis of a large food chemical database: Chemical space, diversity, and complexity. F1000Research.

[50] Domingo-Almenara X., Guijas C., Billings E., Montenegro-Burke J.R., Uritboonthai W., Aisporna A.E., Chen E., Benton H.P., Siuzdak G. (2019). The METLIN small molecule dataset for machine learning-based retention time prediction. Nat. Commun..

[51] Mollica A., Zengin G., Sinan K.I., Marletta M., Pieretti S., Stefanucci A., Etienne O.K., Jekő J., Cziáky Z., Bahadori M.B. (2022). A Study on Chemical Characterization and Biological Abilities of Alstonia boonei Extracts Obtained by Different Techniques. Antioxidants.

[52] Alam M.B., Bajpai V.K., Ra J.S., Lim J.Y., An H., Shukla S., Quan K.T., Khan I., Huh Y.S., Han Y.K. (2019). Anthraquinone-type inhibitor of α-glucosidase enhances glucose uptake by activating an insulin-like signaling pathway in C2C12 myotubes. Food Chem. Toxicol. Int. J. Publ. Br. Ind. Biol. Res. Assoc..

[53] Zhao P., Alam M.B., Lee S.-H. (2019). Protection of UVB-Induced Photoaging by Fuzhuan-Brick Tea Aqueous Extract via MAPKs/Nrf2-Mediated Down-Regulation of MMP-1. Nutrients.

