# Supplementary material for: Antioxidant, Tyrosinase, α-Glucosidase, and Elastase Enzyme Inhibition Activities of Optimized Unripe Ajwa Date Pulp (Phoenix dactylifera) Extracts by Response Surface Methodology
Source: Int J Mol Sci. 2023 Feb 8;24(4):3396. doi: 10.3390/ijms24043396 (PMC9966286; doi:10.3390/ijms24043396)
Supplement: Supplementary file 1 [file ijms-24-03396-s001.zip › ijms-2077125-SI.pdf]

Supplementary data sheet

Table S1: Independent process variables with experimental ranges and levels for heat reflux extraction of URADP.

Input variables	Variable range and levels (coded)						
	unit	Code	-α	-1	0	+1	+α
Ethanol concentration	%	X_1	0	25	50	75	100
Time	min	X_2	15	45	82.5	120	150
Temperature	^{0}C	X_3	40	50	60	70	80

Table S2: Pearson correlation of the TPC, TFC, antioxidants and various enzymes inhibition activities.

	TPC	TFC	DPPH	ABTS	Tyr	Glu
**TFC**	0.943					
	0.001					
**DPPH**	-0.759	-0.905				
	0.048	0.005				
**ABTS**	-0.488	-0.615	0.752			
	0.266	0.142	0.051			
**Tyr**	-0.709	-0.837	0.815	0.734		
	0.075	0.019	0.025	0.060		
**Glu**	-0.702	-0.545	0.385	0.342	0.135	
	0.079	0.206	0.394	0.452	0.774	
**Ela**	0.096	0.321	-0.485	-0.717	-0.455	0.062
	0.839	0.483	0.270	0.070	0.304	0.895

Cell Contents
Pearson correlation,
P-Value
